# Molecular, Structural and Immunological Characterization of Der p 18, a Chitinase-Like House Dust Mite Allergen

**DOI:** 10.1371/journal.pone.0160641

**Published:** 2016-08-22

**Authors:** Yvonne Resch, Katharina Blatt, Ursula Malkus, Christian Fercher, Ines Swoboda, Margit Focke-Tejkl, Kuan-Wei Chen, Susanne Seiberler, Irene Mittermann, Christian Lupinek, Azahara Rodriguez-Dominguez, Petra Zieglmayer, René Zieglmayer, Walter Keller, Vladislav Krzyzanek, Peter Valent, Rudolf Valenta, Susanne Vrtala

**Affiliations:** 1 Division of Immunopathology, Department of Pathophysiology and Allergy Research, Center for Pathophysiology, Infectiology and Immunology, Medical University of Vienna, Vienna, Austria; 2 Division of Hematology and Hemostaseology, Department of Internal Medicine I, Medical University of Vienna, Vienna, Austria; 3 Institute of Medical Physics and Biophysics, University of Münster, Münster, Germany; 4 Division of Structural Biology, Institute of Molecular Biosciences, University of Graz, Graz, Austria; 5 Vienna Challenge Chamber, Vienna, Austria; 6 Institute of Scientific Instruments of the ASCR, Academy of Sciences of the Czech Republic, Brno, Czech Republic; 7 Christian Doppler Laboratory for the Development of Allergen Chips, Medical University of Vienna, Austria; McGill University, CANADA

## Abstract

**Background:**

The house dust mite (HDM) allergen Der p 18 belongs to the glycoside hydrolase family 18 chitinases. The relevance of Der p 18 for house dust mite allergic patients has only been partly investigated.

**Objective:**

To perform a detailed characterization of Der p 18 on a molecular, structural and immunological level.

**Methods:**

Der p 18 was expressed in *E*. *coli*, purified to homogeneity, tested for chitin-binding activity and its secondary structure was analyzed by circular dichroism. Der p 18-specific IgG antibodies were produced in rabbits to localize the allergen in mites using immunogold electron microscopy and to search for cross-reactive allergens in other allergen sources (i.e. mites, crustacea, mollusca and insects). IgE reactivity of rDer p 18 was tested with sera from clinically well characterized HDM-allergic patients (n = 98) and its allergenic activity was analyzed in basophil activation experiments.

**Results:**

Recombinant Der p 18 was expressed and purified as a folded, biologically active protein. It shows weak chitin-binding activity and partial cross-reactivity with Der f 18 from *D*. *farinae* but not with proteins from the other tested allergen sources. The allergen was mainly localized in the peritrophic matrix of the HDM gut and to a lower extent in fecal pellets. Der p 18 reacted with IgE from 10% of mite allergic patients from Austria and showed allergenic activity when tested for basophil activation in Der p 18-sensitized patients.

**Conclusion:**

Der p 18 is a rather genus-specific minor allergen with weak chitin-binding activity but exhibits allergenic activity and therefore should be included in diagnostic test panels for HDM allergy.

## Introduction

HDMs are one of the most important allergen sources worldwide [1,2,3]. Depending on environmental, geographic and climate factors up to 50% of allergic patients are sensitized against HDM allergens [4,5]. Among the house dust mite species, *Dermatophagoides pteronyssinus* and *D*. *farinae* represent the most important allergen sources for allergic patients [6]. HDM-allergic patients’ IgE antibodies show extensive cross-reactivity between *D*. *pteronyssinus* and *D*. *farinae* allergens which is due to high sequence and structural similarities of the allergens [7,8]. More than 30 different house dust mite allergens have been described so far [9,10]. For many of these allergens the frequencies of IgE recognition have been studied in great detail and data regarding their biological functions, allergenic activity and potency are available and this information is important for the development of allergen-specific forms of therapy [11,12,13]. However, much less and controversial information is available for a group of HDM allergens which seem to be associated with chitin [14,15,16]. Among these allergens Der p 23, containing sequences similar to chitin-binding domains, has been identified as a major HDM allergen. [14] Der p 23 is recognized by more than 70% of HDM-allergic patients and shows high allergenic activity. Data regarding the IgE recognition frequency of the chitinase-like group 15 and group 18 HDM allergens are controversial. These allergens also contain a sequence which is homologous to chitin-binding domains [17]. Der f 15 and Der f 18 from *D*. *farinae* have been first described as major allergens for mite allergic dogs with reported IgE binding frequencies of 95% for Der f 15 and 57–77% for Der f 18 [18,19]. IgE recognition frequency data for HDM-allergic patients show large variability. Fifty-four percent of HDM-allergic patients from the Western USA showed IgE reactivity to nDer f 18 [19] whereas Der p 15 and Der p 18 from *D*. *pteronyssinus* were reported to react with IgE antibodies from 70% and 63%, respectively [17]. However, another study reported that only 38% of patients showed IgE reactivity to Der p 15 and Der p 18 [15]. The allergenic activity of the chitinase-like allergens has so far not been studied at all and it is not known if they are linked to certain disease phenotypes such as respiratory or skin allergy. In this context it has been found recently that certain HDM allergens, depending on their localization in the HDM, are associated with certain allergic manifestations (e.g., body-derived allergens: atopic dermatitis; faeces-derived allergens: respiratory allergy) [20].

In this study we re-investigated the frequency of IgE recognition of Der p 18 and studied several hitherto unknown features of this allergen such as allergenic activity, possible association with allergic disease phenotypes and *in situ* localization in the HDM. For this purpose Der p 18 was expressed as folded recombinant protein in *E*. *coli*, purified and used to study IgE recognition frequency and allergenic activity in basophil activation experiments using sera and blood samples of clinically well characterized HDM-allergic patients. Allergen-specific antibody probes were raised to study the cross-reactivity of Der p 18 with Der f 18 and to localize the allergen in HDMs by immunogold electron microscopy. Furthermore, we built a three-dimensional homology model of Der p 18 evaluated by comparisons of secondary structure elements.

## Methods

### Secondary structure model of Der p 18

A 3D-homology model of Der p 18 was generated using the SWISS-MODEL workspace via the ExPASy web server [21]. The proposed chitinase core domain (residues 30–375) was modeled using the PDB template 1waw(A) (human chitotriosidase, sequence identity: 25.1%, E-value: 6*10^−45^, Z-score: -4.042) whereas the PDB template 1dqc(A) (horseshoe crab tachycitin, sequence identity: 20.3%, E-value: 1.4*10^−13^, Z-score: -2.75) was applied on the C-terminal putative chitin-binding domain (residues 404–462). SWISS-MODEL also created a secondary structure alignment between the predicted secondary structure elements of Der p 18 and the secondary structures of both PDB templates which can be used to check the reliability of the model. The core- and the C-terminal domain of Der p 18 were manually arranged in various orientations taking steric constraints into account. These starting conditions were further processed using the web-accessible program ROSETTA DOCK [22] to calculate putative interaction surfaces between both domains by identifying low-energy conformations.

### Expression and purification of recombinant Der p 18

A synthetic gene coding for mature Der p 18 (GenBank accession number Q4JK71) with codons optimized for expression in *E*. *coli* and a hexa-His tag at the 3’ end was *de novo* synthesized and cloned in the *Nde*I/*Eco*RI site of the expression vector pET17b (ATG:biosynthetics, Merzhausen, Germany).

Recombinant Der p 18 was expressed in *E*. *coli* BL21 (DE3) (Stratagene, Santa Clara, CA, USA) as described [23]. After cell lysis [23], the inclusion body fraction containing rDer p 18 was solubilized o/n in 8M urea, 100 mM NaH_2_PO_4_, 10 mM Tris, pH 8 and rDer p 18 was purified by nickel affinity chromatography under denaturing conditions (Quiagen, Hilden, Germany) [24]. Fractions containing rDer p 18 of more than 90% purity were pooled, dialyzed against 10 mM NaH_2_PO_4_, pH 8 and stored at -20°C. The purity of the protein was analyzed by SDS-PAGE under reducing and non-reducing conditions and Coomassie brilliant blue staining [25]. The protein concentration was measured using the BCA Protein Assay Kit (Pierce, Rockford, IL, USA). For control experiments, rDer p 2 was expressed as hexa-histidine-tagged protein in *E*. *coli* and purified as described [26]. Recombinant Der p 5 and Der p 23 were expressed in *E*. *coli* as non-fusions protein and purified to homogeneity by using ion exchange chromatography, as previously described [14,23].

### Biochemical and biophysical characterization of rDer p 18

#### Liquid chromatography-ion trap mass spectrometry

Protein bands were excised from the rDer p 18 preparations which had been separated by SDS-PAGE and were digested using the Trypsin Profile IGD Kit (Sigma-Aldrich, St. Louis, MO, USA). The Nano LC-ESI MS/MS data were acquired and analysed as described [27] except that generated peak lists were searched against the Swiss Prot databank using MASCOT (Matrix Science) search engine.

#### Circular dichroism spectroscopy

Far UV CD spectra of rDer p 18 dissolved in 10 mM NaH_2_PO_4_, pH 8 (final protein concentration of 0.1 mg/ml) were collected on a Jasco J-810 spectropolarimeter (Japan Spectroscopic Co., Tokyo, Japan) using a 1mm path length quartz cuvette. Measurements were done between 250 to 190 nm, with 0.5 nm resolution at a scanning speed of 50 nm/min. Three independent measurements were recorded and averaged for each spectral point. The final spectra were baseline corrected by subtracting the corresponding buffer spectrum. Results were expressed as the mean residue ellipticity [θ] at a given wavelength. The secondary structure estimation program CDSSTR (reference data set 7) on the DichroWeb server [28] was used to calculate the secondary structure of the protein.

#### Chitin-binding assay

Chitin-binding assays were performed as previously described with slight modifications [16]. Five mg of chitin from shrimp shells (Sigma St. Louis, MO, USA) or 50 μl of chitin beads (New England Biolabs, Ipswich, MA, USA) were incubated with 150 μg of HDM extract or 10 μg of Der p 18, rDer p 15, wheat germ agglutinin (WGA, positive control), or rDer p 5 (negative control) in 400 μl of 50 mM Tris pH 8, 100 mM NaCl at RT for one hour by orbital shaking, following centrifugation. Chitin pellets were washed five times with buffer and bound proteins were eluted with 4x SDS-sample buffer [62 mM Tris, 200 mM SDS, 10% (v/v) glycerol, 5% (v/v) β-mercaptoethanol, 2.5% (v/v) bromphenolblue dissolved in 1% methanol, pH 6.8] at 95°C for 5 min. Supernatants were concentrated by the SpeedVac UniVapo 150 ECH (Uniequip, Planegg, Germany). Purified proteins (5 μg aliquots) or HDM extract (50 μg aliquots), supernatant and the eluted proteins from the pellet were analysed by 12.5% SDS-PAGE and Coomassie Blue staining.

### Der p 18-specific antibodies

A rabbit antiserum was raised against rDer p 18 by immunizing a rabbit with the purified protein (3 times à 200 μg, using once Freund's complete and twice Freund's incomplete adjuvant) by the company Charles River (Kisslegg, Germany).

### Preparation of protein extracts from mites, seafood and wasps and immunoblotting

Aliquots of 0.3 g of the different purified mite species (i.e., whole body preparations) (*D*. *pteronyssinus*, *D*. *farinae*, *Blomia tropicalis*; Allergon, Vällinge, Sweden) and *D*. *pteronyssinus* feces (kind contribution from Fernández-Caldas E, Immunotek S. L., Madrid, Spain) were homogenized in 5 ml 4x SDS-sample buffer or 5 ml 1 x phosphate buffered saline, pH 7 containing 1 μg/ml phenylmethylsulfonyl fluoride, respectively, using an Ultra-Turrax T25 Basic disperser (IKA, Staufen, Germany). The homogenates were incubated over night at 4°C and the insoluble fraction was removed by centrifugation (20 min, 4000xg, 4°C). The protein content of the different extracts was analyzed by SDS-PAGE and Coomassie Brilliant Blue staining.

Extracts from Vespula spp. and locally purchased fresh shrimp, lobster, squid and snail were prepared by homogenizing 2 g of tissue samples in 10 ml 4x SDS-sample buffer using an Ultra-Turrax T25 Basic disperser (IKA). The samples were incubated for 15 min at 95°C before a second homogenization event. Debris was removed by centrifugation at 4000xg for 10 min at 4°C and the collected supernatant was stored at –20°C until use.

For immunoblotting, equal amounts of the extracts were separated by 12.5% SDS-PAGE and blotted onto nitrocellulose membranes (Schleicher & Schuell, Dassel, Germany). The membranes were blocked two times for 5 min and once for 30 min in buffer B [40 mM NaH_2_PO_4_, 0.6 mM Na_2_HPO_4_, 0.5% (v/v) Tween-20, 0.5% (w/v) BSA and 0.05% (w/v) sodium azide] and incubated with rabbit anti-rDer p 2 antibodies, anti-rDer p 18 antibodies or normal rabbit antibodies, diluted 1: 5 000 or 1: 10 000 in buffer B o/n at 4°C. After washing, the nitrocellulose membranes were incubated with ^125^I-labeled donkey anti-rabbit antibodies (Perkin Elmer, Boston, MA, USA) and bound antibodies were detected by autoradiography (Kodak XOMAT film, Kodak, Heidelberg, Germany) [29].

For inhibition experiments, rabbit anti-Der p 18 antibodies or normal rabbit antibodies (1: 50 000 dilution) were pre-incubated with 200 μg of *D*. *pteronyssinus*, *D*. *farinae* extracts, 20 μg of Der p 18, Der p 15, or, for control purposes, with 20 μg of BSA, o/n at 4°C. The preincubated serum samples were then exposed to nitrocellulose-bound *D*. *pteronyssinus* or *D*. *farinae* extract o/n at 4°C and bound IgG Abs were detected as described above.

### Immunogold electron microscopy

Der p 18-specific IgG antibodies were purified from the serum of a Der p 18-immunized rabbit using a protein G column (ImmunoPure IgG Purification Kit, Pierce). IgG antibodies purified from the pre-immune serum were used as control. The purified Ig fractions were used to detect Der p 18 in the mite body and feces by immunogold electron microscopy [23].

### IgE reactivity and allergenic activity of rDer p 18

Sera analyzed in this study were residual samples obtained from HDM-allergic adult Austrian patients (S1 Table, sera #1–91; median age: 25 years, range: 18–59 years) before immunotherapy [30]. These patients suffered from HDM-induced allergic rhinitis with or without asthma or atopic dermatitis (S1 Table). Patients showed positive skin prick test reactions (SPT) to *D*. *pteronyssinus* extract (wheal diameter ≥3 mm larger than saline control, median: 10 mm, range: 3–20 mm) and *D*. *pteronyssinus*-specific IgE levels above 0.7 kU/L (median: 10.1 kU/L, range: 1.11–100 kU/L) (S1 Table). The frequency of IgE recognition of Der p allergens was determined by ImmunoCAP ISAC technology (Thermofisher, Uppsala, Sweden) in sera #1-#91 [31].

In addition, sera and fresh blood samples were obtained from HDM-allergic adult patients (sera #92–100) for dot blot, western blot inhibitions and basophil activation testing, who according to a case history indicative for HDM allergy, positive SPT and *D*. *pteronyssinus*-specific IgE suffered from HDM allergy. Sera from these HDM-allergic individuals (sera #92–100) and from a non-allergic individual were tested for IgE reactivitiy to rDer p 2, rDer p 18 and Der p 23 in a non-denaturing dot blot assay as described [32]. For the determination of the allergenic activity of rDer p 18, peripheral blood from four HDM-allergic patients (sera #92–95) containing Der p 18-specific IgE antibodies were incubated with serial dilutions of Der p 18, and for control purposes with Der p 2 (0.0001 μg/ml to 10 μg/ml). The up-regulation of CD203c expression on basophils was determined as described [33].

### IgE immunoblot inhibition assay

For IgE inhibition experiments, nitrocellulose-blotted *D*. *pteronyssinus* extract was incubated with sera from two mite allergic patients with IgE reactivity to Der p 18 which had been diluted 1:10 in buffer B and were pre-incubated with 10 μg/ml rDer p 18 or BSA o/n at 4°C. The pre-incubated serum samples were then exposed to the nitrocellulose-strips o/n at 4°C and bound IgE antibodies were detected with ^125^I-labeled anti-human IgE antibodies (Demeditec Diagnostics, Kiel, Germany) and visualized by autoradiography (Kodak XOMAT film).

### Ethical considerations

Residual serum samples #1–91 which had been obtained in the course of routine allergy diagnosis when patients were enrolled for a HDM immunotherapy study [30] (Clinicaltrials.gov identifier: NCT01644617) were analysed for IgE reactivity to purified HDM allergens. This retrospective and anonymized analysis of allergen-specific IgE antibodies in the residual serum samples was performed with permission of the Ethics Committee of the Medical University of Vienna (EK 1641/2014). Fresh blood samples and sera from the HDM-allergic patients (#92–100) and the non-allergic individual were obtained from the subjects after written informed consent was obtained and analyzed for IgE reactivity and basophil activation with permission of the Ethics Committee of the Medical University of Vienna (EK 1641/2014).

## Results

### Building a protein structure homology model of Der p 18

**Fig 1:**
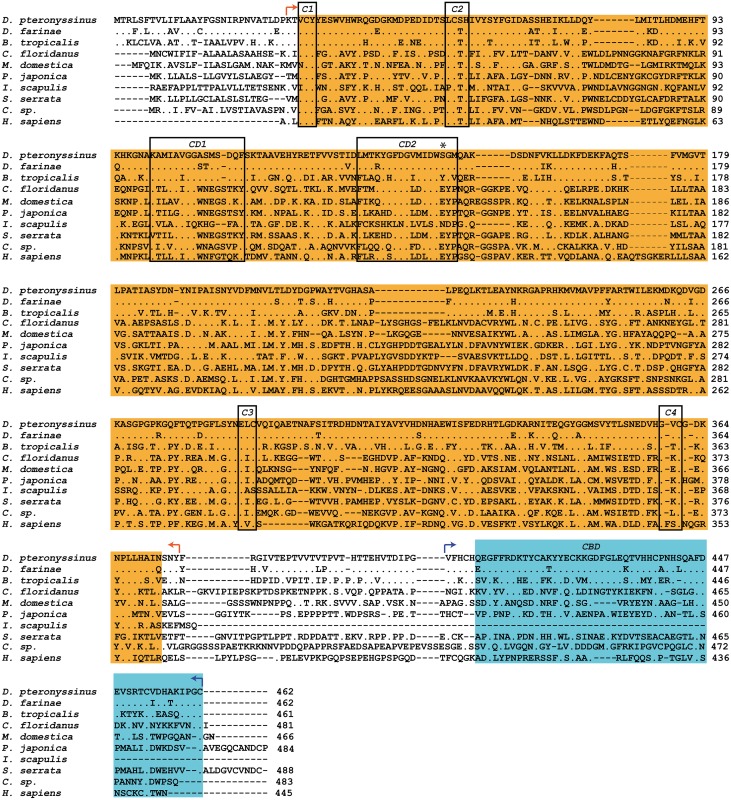
Multiple alignment of Der p 18 amino acid sequence with homologous proteins from other mites, insects, crustacea and man. Species and GenBank accession numbers: *Dermatophagoides pteronyssinus* (house dust mite, Der p 18, AAY84563.1), *Dermatophagoides farinae* (house dust mite, Der f 18, AAM19082.1), *Blomia tropicalis* (storage mite, Blo t 18, AAQ24549.1), *Camponotus floridanus* (carpenter ant, EFN71329.1), *Musca domestica* (housefly, ABI29879.1), *Pandalopsis japonica* (shrimp, AFC60661.1), *Ixodes scapularis* (tick, XP_002404708.1), *Scylla serrata* (crab, ABY85409.1), *Chelonus sp*. (wasp, AAA61639.1), *Homo sapiens* (human, 1WAW_A). Marked in orange are the chitinase core domain including the conserved cysteines of the catalytic region (C1-C4) and the putative catalytic domains (CD1, CD2: the asterisk indicates the position of the glutamic acid that determines the presence of enzymatic activity). The putative chitin-binding domain (CBD) is highlighted in blue. Red and blue arrows indicate the borders of the segments which were used to create the structural model of Der p 18 (see Fig 2). Amino acids identical to those of Der p 18 are indicated by dots; dashes represent gaps.

A comparison of the amino acid sequence of Der p 18 (AAY84563.1) with sequences deposited in the NCBI database using the Basic Local Alignment Search Tool (BLAST) indicates that Der p 18 belongs to the GH 18 (glycosyl hydrolase, family 18) chitinase-like superfamily and shows a high sequence homology with Der f 18 from *D*. *farinae* (88%) as also noted earlier [17]. In addition we found a significant sequence identity of 59% with a Blo t chitinase-like allergen from *B*. *tropicalis* (Fig 1, Table 1). The sequences from GH 18 chitinase-like proteins from seven other species (i.e. ant, fly, shrimp, tick, crab, wasp and human) showed a much lower sequence identity with Der p 18 ranging from 30%-21% (Fig 1, Table 1). The sequence of Der p 15, which according to sequence homology, also belongs to the GH 18 chitinase-like proteins shows only 27% sequence identity to Der p 18 (S1 Fig). The multiple sequence alignment of Der p 18 with the other GH 18 chitinase-like proteins shows that the architecture of these proteins consists of a proposed chitinase core domain and a putative C-terminal chitin-binding domain (Fig 1). Regions with high sequence conservation can be found in the chitinase core domain, such as the four conserved cysteine residues (C1-4) and the two catalytic domains (CD1, CD2), with the signature sequence FDxxDxDxE of CD2 (Fig 1, framed boxes). The glutamate residue (E) at position 148, which is thought to be essential for the chitinase activity is absent in Der p 18 and the other group 18 mite allergens, indicating that they belong to the non-enzymatically-active group of chitinase-like proteins. Like all the aligned sequences of Fig 1, except the chitinase of *I*. *scapulis* (tick), Der p 18 contains a C-terminal putative chitin-binding domain (CBM 14, pfam01607), which is often found in peritrophic matrix proteins of insects and animal chitinases [34,35,36]. Der p 18 contains 5 of the 6 conserved cysteines, which are characteristic for this domain. This domain can be also found in Der p 15 but in Der p 15, the chitinase core domain is connected with the putative chitin-binding domain via a longer stretch containing a repeated sequence which is rich in serine, threonine and proline (S1 Fig). Based on the overall conserved architecture of the GH 18 chitinase-like proteins we made an attempt to build homology models of the proposed chitinase core domain and the putative C-terminal chitin-binding domain of Der p 18 with SWISS-MODEL. The signal sequence at the N-terminus (residues 1–29) and a 29 residue connecting sequence between both domains (residues 375–404) were not processed due to the absence of an appropriate homologous sequence in the PDB (Protein Data Bank).

**Table 1:** Percentages of amino acid sequence identities among chitinases and chitinase-like proteins from mites, insects, crustaceans and human^1^.

	***D*. *pteronyssinus***	***D*. *farinae***	***B*. *tropicalis***	***C*. *floridanus***	***M*. *domestica***	***P*. *japonica***	***I*. *scapularis***	***S*. *serrata***	***C*. *sp*.**	***H*. *sapiens***
***D*. *pteronyssinus***	100	88	59	30	28	28	27	25	24	21
***D*. *farinae***		100	61	30	27	28	26	24	25	20
***B*. *tropicalis***			100	29	26	25	28	26	27	23
***C*. *floridanus***				100	42	40	33	41	47	39
***M*. *domestica***					100	36	30	36	41	35
***P*. *japonica***						100	31	57	38	35
***I*. *scapularis***							100	33	33	28
***S*. *serrata***								100	39	37
***C*. *sp*.**									100	36
***H*. *sapiens***										100

^1^*Dermatophagoides pteronyssinus* (house dust mite, Der p 18, AAY84563.1), *Dermatophagoides farinae* (house dust mite, Der f 18, AAM19082.1), *Blomia tropicalis* (storage mite, Blo t 18, AAQ24549.1), *Camponotus floridanus* (carpenter ant, EFN71329.1), *Musca domestica* (housefly, ABI29879.1), *Pandalopsis japonica* (shrimp, AFC60661.1), *Ixodes scapularis* (tick, XP_002404708.1), *Scylla serrata* (crab, ABY85409.1), *Chelonus sp*. (wasp, AAA61639.1), *Homo sapiens* (human, 1WAW_A). The percentage of identity is shown.

**Fig 2:**
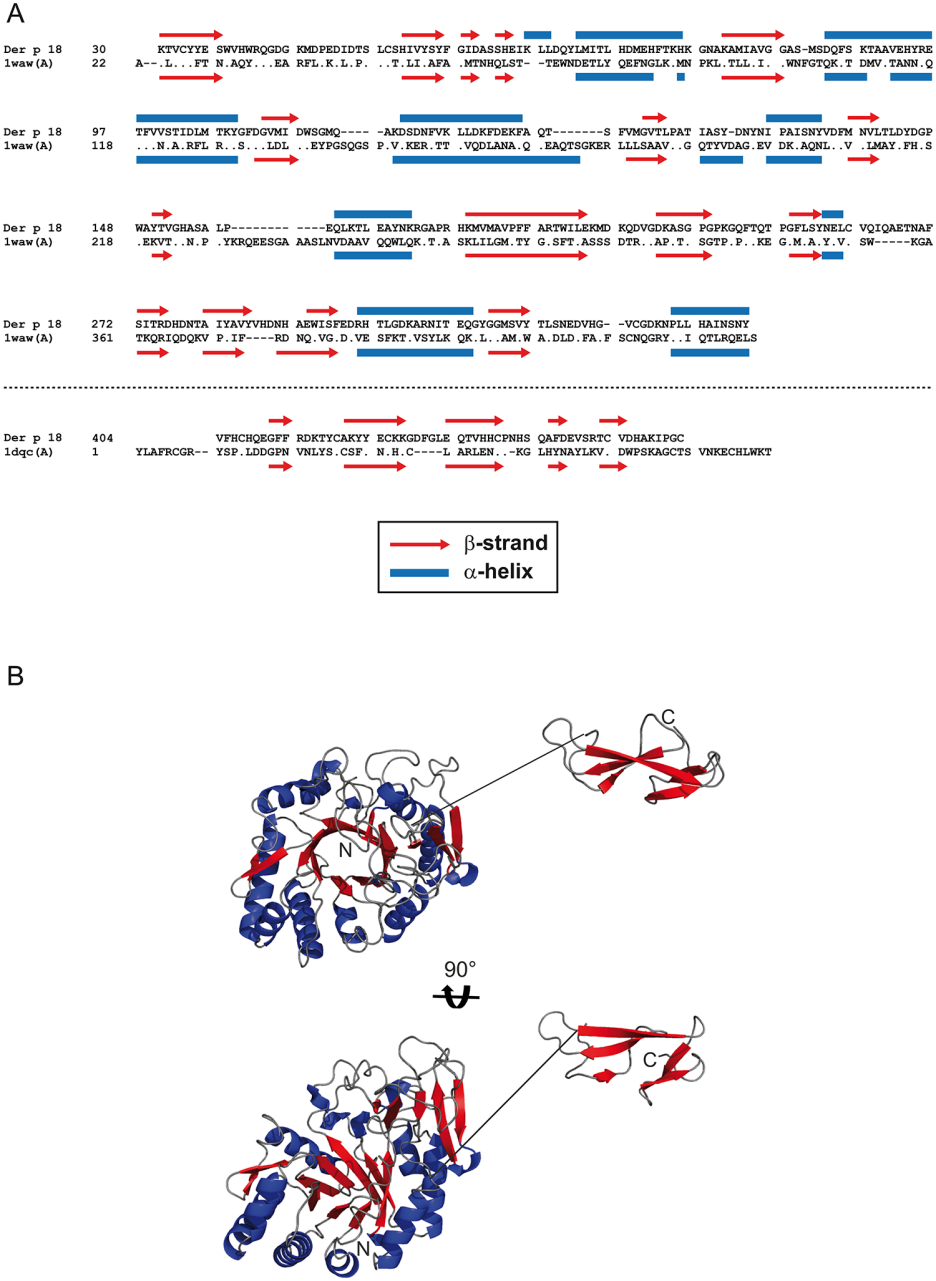
Structural alignment. (A) Alignment of predicted secondary structure elements of Der p 18 with the secondary structure of human chitinase (1wawA) for portions of human chitinase with known three-dimensional structure and of the Der p 18 putative chitin-binding domain with tachycitin (1dqcA) as created with the SWISS-MODEL program. Identical amino acids are indicated by dots, dashes represent gaps and similar secondary structure elements (β-strands, α-helices) are marked. (B) Structural model of Der p 18 generated by the SWISS-MODEL program. The 29-amino-acid connecting sequence is indicated by a continuous line.

The alignment of the predicted secondary structure elements of Der p 18 with the secondary structures found in the three-dimensional structures of the templates (1waw, 1dqc) (Fig 2A) revealed a high degree of similarity regarding the secondary structure arrangement of Der p 18 and both template structures (1waw, 1dqc) within the modeled range. Hence, the models might give a good representation of the actual backbone arrangement of Der p 18 despite low sequence identity in both cases.

Attempts to find the most probable orientation upon energy minimization calculations between the (α/β)_8_-TIM barrel- of the proposed chitinase core domain and the C-terminal putative chitin-binding domain with ROSETTA DOCK [22] did not yield any low-energy complexes between the two domains (data not shown). Therefore it is likely that the domains adopt independent orientations in solution, but still form a functional unit due to the spatial proximity of the two domains (Fig 2B).

### Expression, purification and characterization of folded biologically active recombinant Der p 18

**Fig 3:**
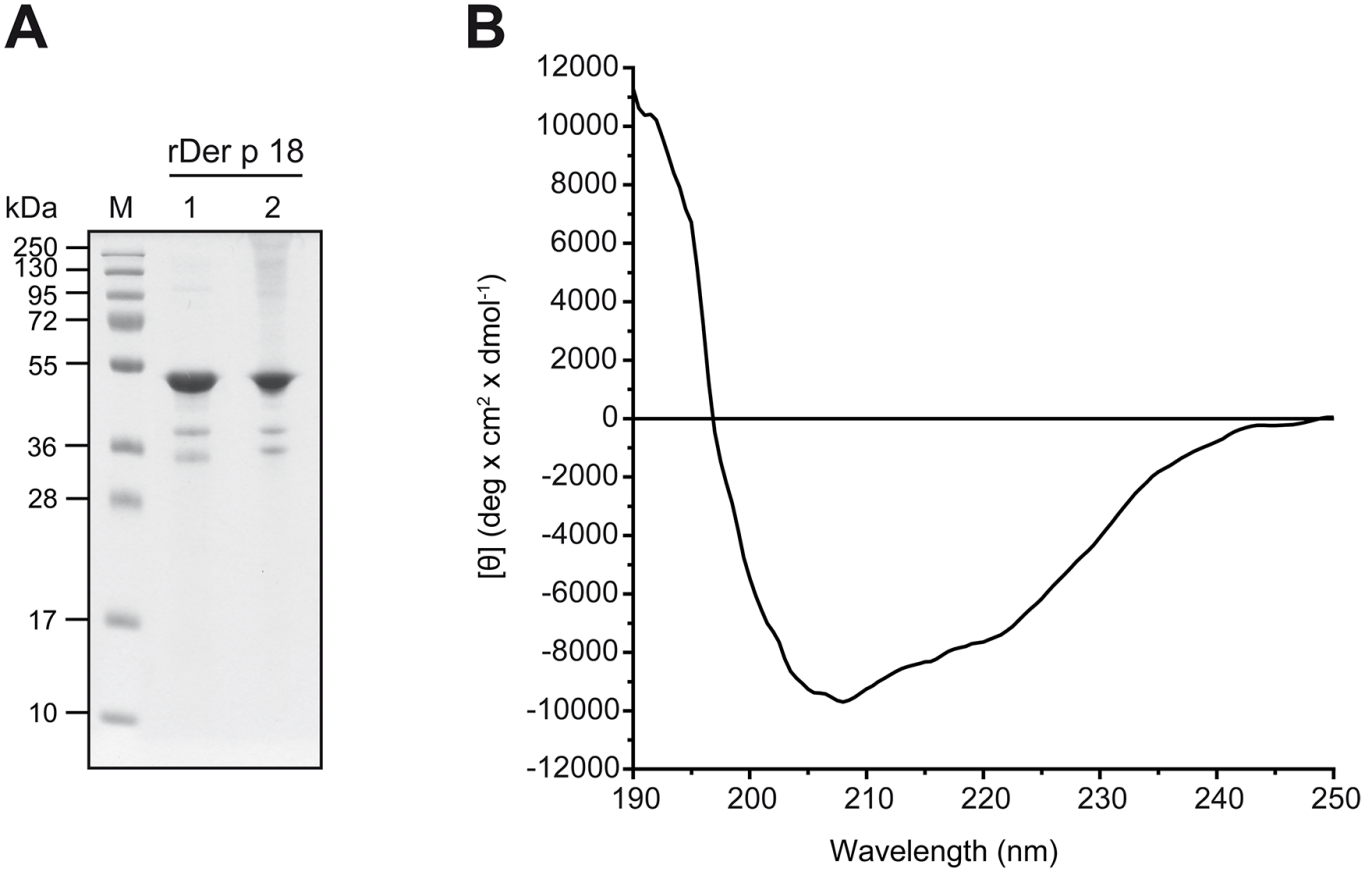
Characterization of purified rDer p 18. (A) An aliquot of 3 μg of rDer p 18 was separated by SDS-PAGE under reducing (1) and non-reducing (2) conditions and stained with Coomassie brilliant blue. M, molecular weight marker. (B) Far UV CD analysis of rDer p 18. The graph represents the mean residue ellipticity (θ, y-axis) at a given wavelength (190–250 nm, x-axis).

Recombinant Der p 18 was expressed in *E*. *coli* and purified from the inclusion body fraction. After solubilization, around 7 mg of protein/liter bacterial culture was purified by Ni-affinity chromatography. The purified and refolded protein migrated at a molecular weight of 51 kDa in SDS-PAGE (Fig 3A, lane 1), which corresponds to the size calculated from its aa sequence (51.03 kDa). Two additional weak bands at 35 kDa and 40 kDa observed in the rDer p 18 preparation were identified as fragments by mass spectrometry (data not shown). Under non-reducing conditions (Fig 3A, lane 2) a small portion of rDer p 18 formed high molecular weight aggregates.

The far-UV CD spectrum of rDer p 18 (Fig 3B) recorded at room temperature showed a maximum at 193 nm and two minima at 208 nm and 222 nm, indicating that the recombinant allergen is folded. Calculating the secondary structure content using the program CDSSTR yielded 18% α-helix, 22% β-sheet, 18% β-turn and 42% random coil (normalized root-mean-square deviation: 0.034). The equal ratio of α-helix and β-sheet content is in accordance with published three dimensional structures of chitinases, which show that they contain a (α/β)_8_-TIM barrel structure [37].

**Fig 4:**
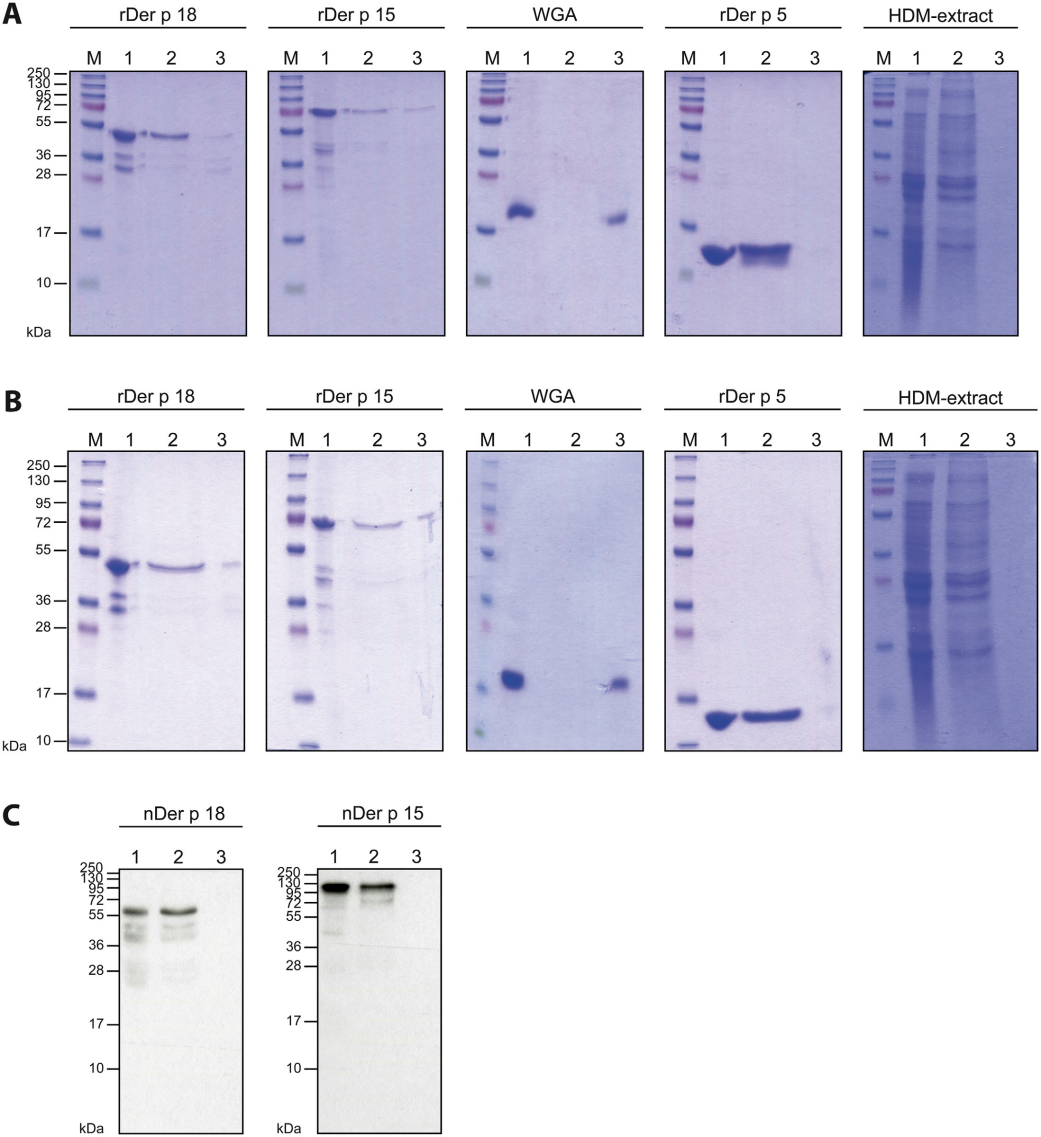
Chitin-binding activity of Der p 18. Coomassie-stained SDS-PAGE containing aliquots of rDer p 18, rDer p 15, WGA (positive control) and rDer p 5 (negative control) or HDM extract (lanes 1), the supernatants of these proteins (lanes 2) and proteins eluted from the chitin/chitin beads (lanes 3). The molecular weight marker is shown in lanes M. (C) Nitrocellulose-blotted samples of the experiment performed with HDM-extract and chitin beads (B) were incubated with rabbit anti-Der p 18 or anti-Der p 15 antibodies.

To investigate the ability of Der p 18 to bind chitin, chitin-binding assays were performed with chitin powder from shrimp shells (Fig 4A) as well as with chitin beads (Fig 4B), which showed comparable results. Recombinant Der p 18 as well as rDer p 15 bound weakly to chitin, whereas rDer p 5, which lacks a chitin-binding domain, failed to bind. The chitin-binding protein WGA used as positive control, strongly reacted with chitin (Fig 4A and 4B). When the same chitin-binding assay was performed with a HDM extract, we could not detect proteins which bound to chitin by Coomassie staining (Fig 4A and 4B). Natural Der p 18 and nDer p 15, although present in the HDM extract were not found even with antibody probes in the fraction eluted from chitin (Fig 4C).

### Limited cross-reactivity of rabbit anti-Der p 18 antibodies indicates that Der p 18 represents a genus-specific allergen

**Fig 5:**
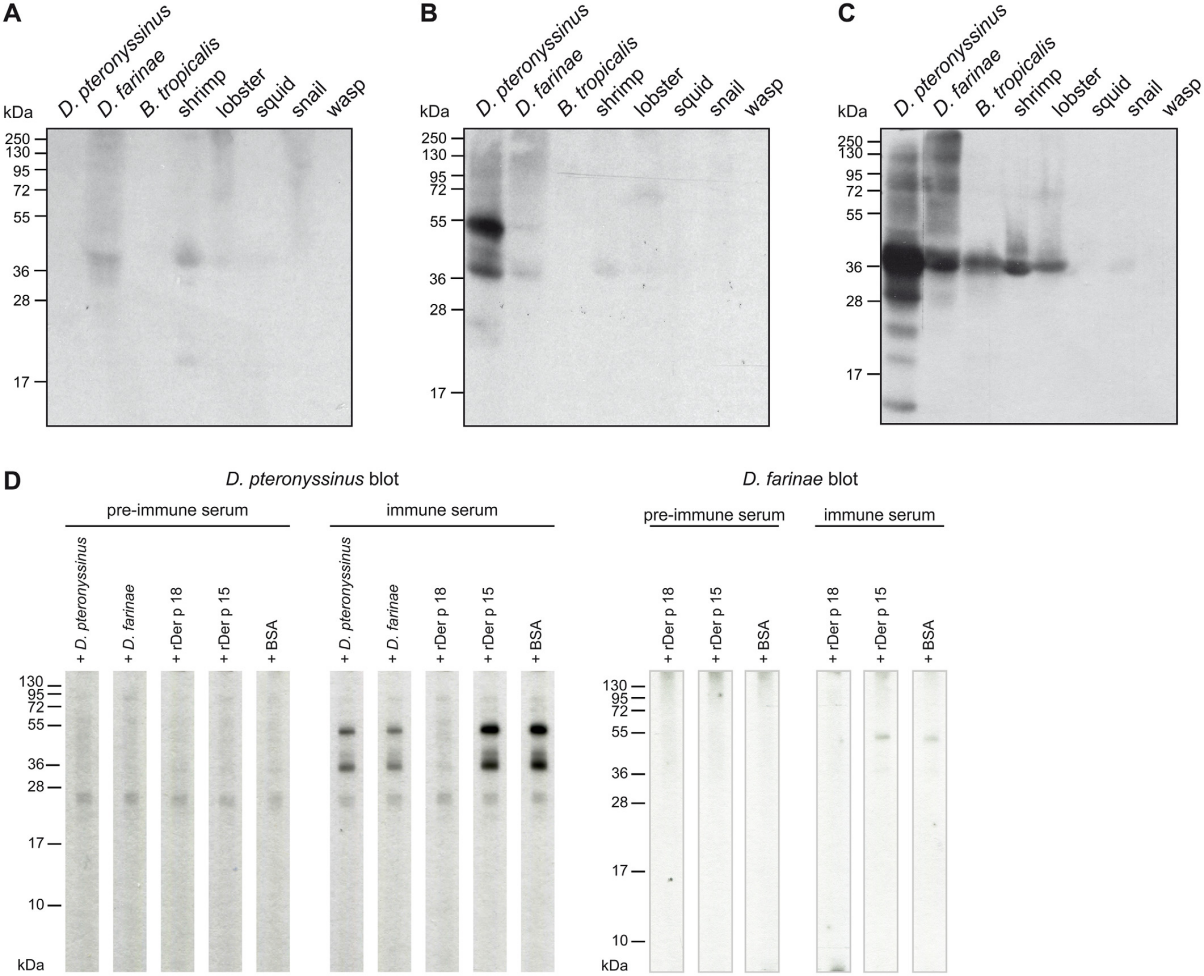
Cross-reactivity of rabbit anti-rDer p 18 IgG antibodies with proteins from other mites, crustacea, mollusca and insects. Blots containing extracts from *Dermatophagoides pteronyssinus*, *Dermatophagoides farinae*, *Blomia tropicalis*, shrimp, lobster, snail and wasp which had been separated by SDS-PAGE were incubated with normal rabbit antibodies before immunization (A), with rabbit anti-rDer p 18 (B) or with rabbit anti-rDer p 10 antibodies (C). (D) Inhibition of IgG reactivity to blotted nDer p 18 and nDer f 18. Nitrocellulose-blotted *D*. *pteronyssinus* (left panel) and *D*. *farinae* (right panel) extracts were incubated with a rabbit anti-Der p 18 pre-immune serum or immune serum, which had been pre-incubated with *D*. *pteronyssinus* extract, *D*. *farinae* extract, rDer p 18, rDer p 15 or BSA. Molecular weights (kDa) are shown at the margins.

We used rabbit anti-Der p 18 antibodies to search for cross-reactive proteins in various species, including those where Der p 18-homologous proteins were identified by BLAST analysis (see Fig 1). The rabbit IgG antibodies strongly reacted at 51 kDa and 40 kDa with *D*. *pteronyssinus*, but only very weakly with Der f 18 from *D*. *farinae*, although the sequence identity between Der p 18 and Der f 18 is very high (Table 1: 88%) (Fig 5B). Rabbit anti-Der p 10 antibodies strongly cross-reacted with tropomyosins in the blotted *D*. *farinae* extract and in several other tested extracts (e.g., *Blomia*, shrimp, lobster) (Fig 5C). Preadsorption of rabbit anti-Der p 18 antibodies with *D*. *pteronyssinus* as well as *D*. *farinae* inhibited partially the IgG binding to Der p 18 in the blotted *D*. *pteronyssinus* extract (Fig 5D), indicating that Der f 18 shares epitopes with Der p 18 that are recognized by the rabbit anti-Der p 18 antibodies. Furthermore, preadsorption of rabbit anti-Derp 18 antibodies with rDer p 18 completely inhibited the weak binding to nDer f 18 in *D*. *farinae* extract (Fig 5D). Consistent with the low sequence homology seen in the alignment (Fig 1, Table 1), Der p 18-specific Abs did not react with the tropical mite *B*. *tropicalis*, crustacean, mollusca and insects at 51 kDa (Fig 5B). Our results thus indicate that Der p 18 is a genus-specific allergen.

### Der p 18 is localized in the peritrophic matrix of *D*. *pteronyssinus*, but is almost absent in feces

**Fig 6:**
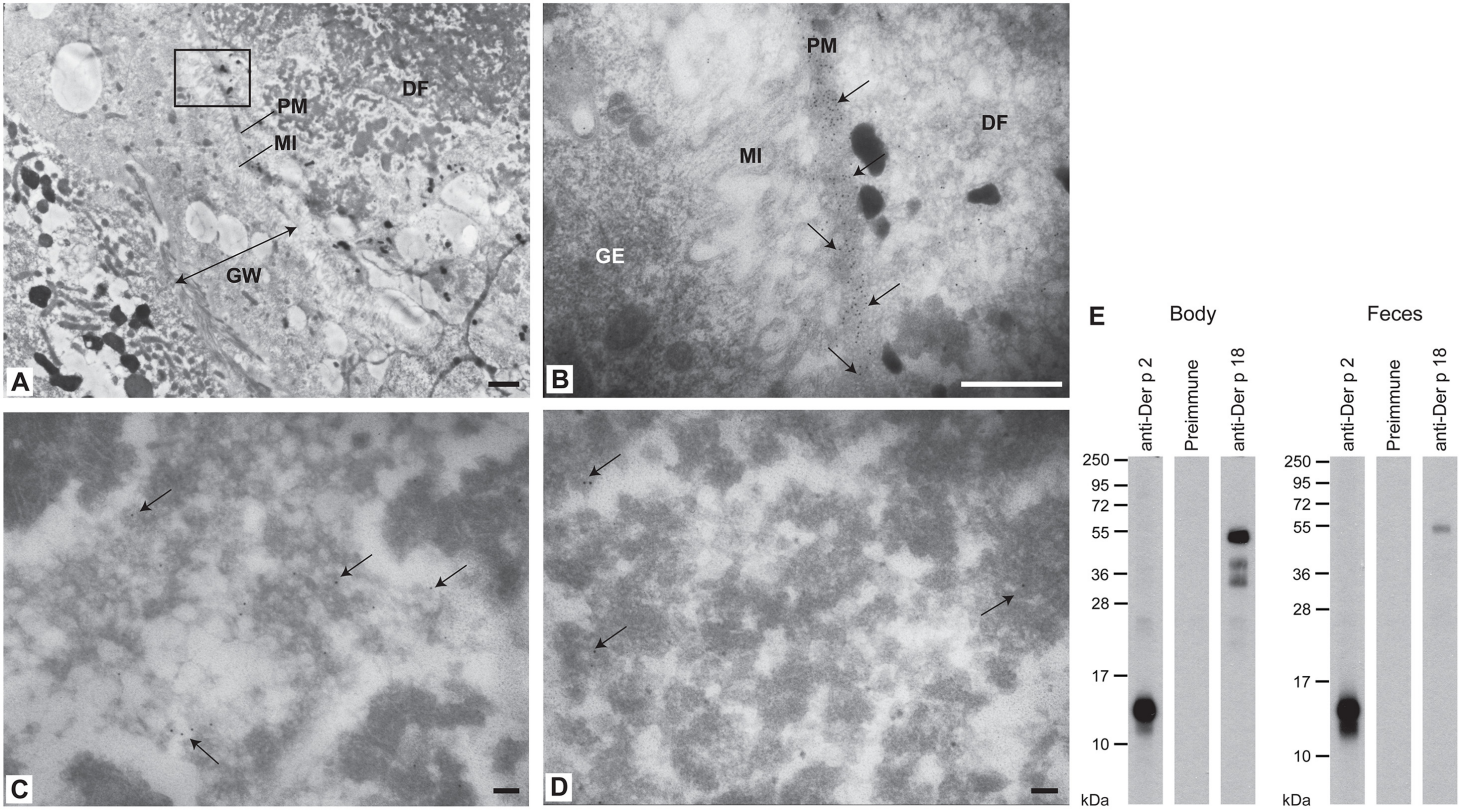
Localization of Der p 18 in *D*. *pteronyssinus* by immunogold electron microscopy and immunoblotting. (A) Gut overview. The section marked by the rectangle is shown in (B) at higher magnification. (B) Specific labeling of Der p 18 in the peritrophic matrix. (C) Localization of Der p 18 in fecal particles after incubation with anti-Der p 18 antibodies or (D) with pre-immune serum. Arrows indicate gold particles. Bars: A, 1 μm; B, 500 nm; C, 100 nm; D, 100 nm. DF, digested food; GE, gut epithelium. GW, gut wall; MI, microvilli; PM, peritrophic matrix. (E) Nitrocellulose-blotted extracts of mite bodies and feces were incubated with rabbit anti-rDer p 18, anti-rDer p 2 antibodies or normal rabbit antibodies.

Next we investigated the *in situ* localization of Der p 18 in HDMs using immunogold electron microscopy. Fig 6A gives an overview of the anterior midgut showing the gut wall, microvilli, peritrophic matrix as well as digested food. At high magnification, Der p 18 was found in the peritrophic matrix of the gut which surrounds the digested food (Fig 6B). Only few gold particles were found in fecal particles after incubation with anti-Der p 18 Abs (Fig 6C), but this reactivity was not specific because it was also found with the rabbit pre-immune antibodies (Fig 6D). In Western blot experiments, anti-Der p 18-specific rabbit antibodies mainly detected the allergen in the mite body extract but only weakly in the feces extract (Fig 6E). The pre-immune serum did not show any binding to the body and feces extract. Der p 2 was detected in the body and feces extract (Fig 6E).

### Der p 18 binds IgE from 10% of HDM-allergic individuals and has similar IgE reactivity as natural Der p 18

**Fig 7:**
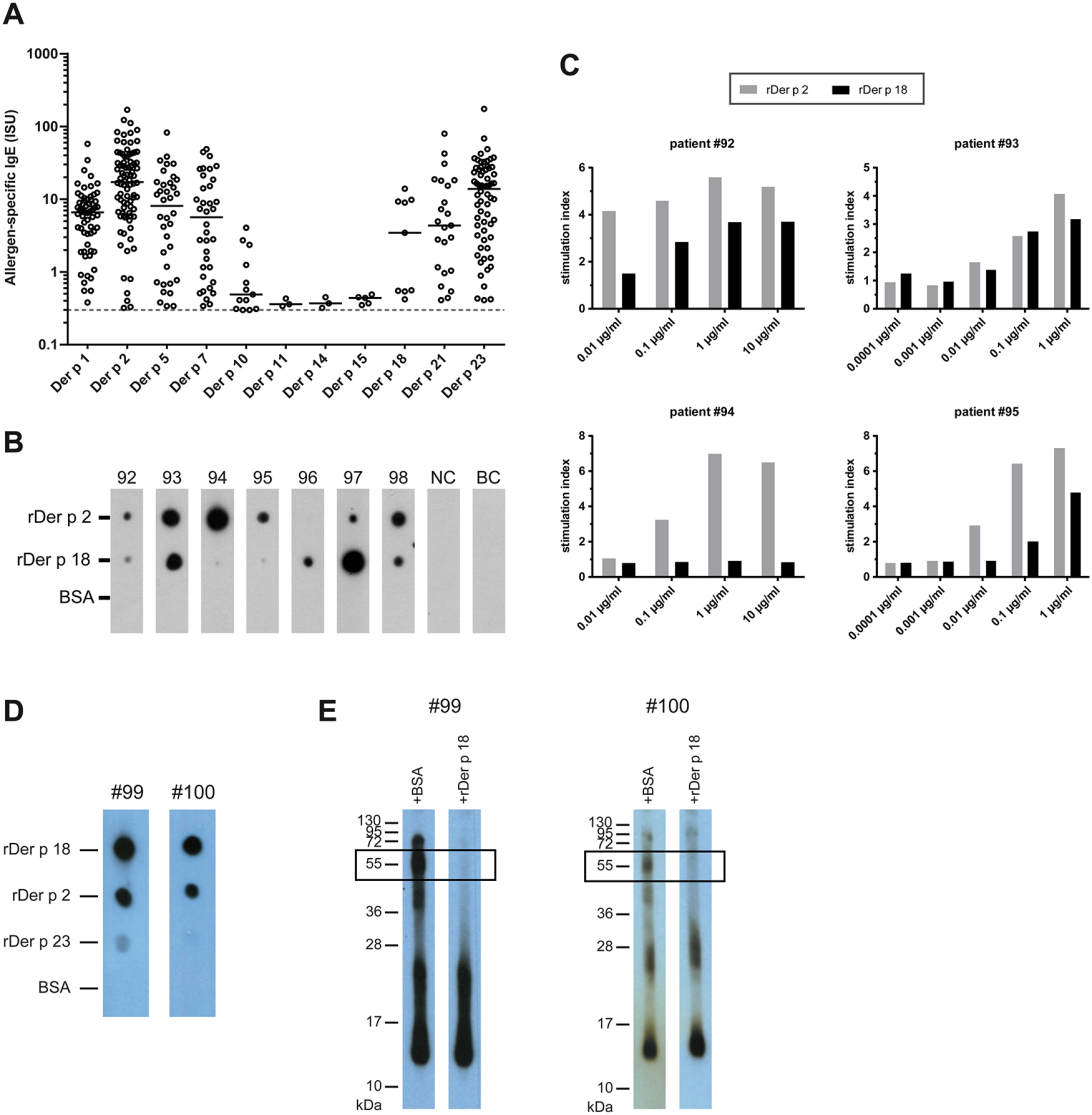
IgE reactivity and allergenic activity of rDer p 18. (A) IgE levels (y-axis: ISU ISAC standardized units) specific for a panel of HDM allergens (x-axis: Der p 1, Der p 2, Der p 5, Der p 7, Der p 10, Der p 11, Der p 14, Der p 15, Der p 18, Der p 21 and Der p 23) in HDM-allergic patients. (B) Dot-blotted rDer p 2, rDer p 18 and BSA were tested for IgE reactivity with sera from 7 HDM-allergic patients positive to rDer p 18 (#92–98), serum of a non-allergic person (NC) and with buffer (BC). Bound IgE Abs were detected with ^125^I-labeled anti-human IgE Abs and visualized by autoradiography. (C) Up-regulation of CD203c expression was determined by FACS analysis after incubation of basophils from 4 HDM-allergic patients (#92–95) with increasing concentrations of rDer p 2 or rDer p 18 (x-axes) and displayed as stimulation index on the y-axes. (D) Recombinant Der p 18, rDer p 2, rDer p 23 and BSA were dotted onto nitrocellulose strips and incubated with sera from Der p 18-positive patients (#99, #100). Bound IgE antibodies were detected with ^125^I-labeled anti-human antibodies and visualized by autoradiography. (E) Inhibition of IgE reactivity to blotted nDer p 18 by rDer p 18. Nitrocellulose-blotted *D*. *pteronyssinus* extract was incubated with sera from Der p 18-sensitized patients (#99 and #100), which had been pre-incubated with rDer p 18, or for control purposes, with BSA. Molecular weights (kDa) were shown at the margins.

We then studied the prevalence of IgE binding to 11 HDM allergens including rDer p 18 with sera from 91 clinically well characterized HDM-allergic patients by ImmunoCAP ISAC technology (Fig 7A, S1 Table). The frequencies of IgE recognition were as follows: Der p 1: 67% (61/91), Der p 2: 92% (84/91), Der p 5: 40% (36/91), Der p 7: 40% (36/91), Der p 10: 14% (13/91), Der p 11: 3% (3/91), Der p 14: 3% (3/91), Der p 15: 6% (5/91), Der p 18: 10% (9/91), Der p 21: 25% (23/91) and Der p 23: 70% (64/91).

In the Der p 18 positive sera (n = 9), the levels of Der p 18-specific IgE antibodies ranged from 0.42 ISU to 13.96 ISU (median: 3.45 ISU), compared to a median IgE binding of 17.20 ISU for the major allergen, Der p 2 (range: 0.32–170.07 ISU). The IgE levels to Der p 18 were much higher compared to that of the other spotted high-molecular-weight allergens Der p 11, Der p 14 and Der p 15 (median: 0.36 ISU, 0.37 ISU and 0.44 ISU, respectively) (Fig 7A). The frequency of IgE recognition of Der p 18 was similar in patients with respiratory and skin manifestations. Among patients who suffered from asthma in addition to allergic rhinitis (n = 20), Der p 18 was recognized by 15%. Likewise, 15% of patients with atopic dermatitis (n = 13) reacted with Der p 18.

Fig 7B shows the intensity of IgE reactivity of sera from seven representative Der p 18-positive patients to rDer p 18 in comparison to rDer p 2. Five of these patients showed comparable or even stronger IgE reactivity to rDer p 18 than to Der p 2. Patient #96 was remarkable because the serum showed IgE reactivity only to Der p 18 when tested for the complete panel of HDM allergens on the chip (data not shown). This patient suffered from allergic rhinoconjunctivitis and atopic dermatitis. Serum from a non-allergic individual and the buffer control did not show IgE-reactivity to any of the proteins. None of the sera showed IgE reactivity to BSA (Fig 7B).

When sera from two Der p 18-positive patients (Fig 7D, #99, #100) were used for IgE inhibition experiments, we found that preincubation of the sera with rDer p 18 inhibited IgE binding to natural Der p 18 completely, indicating that IgE epitopes of the natural protein are correctly represented by the recombinant protein (Fig 7E).

### rDer p 18 induces dose-dependent basophil activation

The allergenic activity of rDer p 18 was analysed using basophils from four HDM-allergic individuals, who had shown IgE reactivity to Der p 18 (see patients #92–95, Fig 7B). The up-regulation of CD203c expression was measured after incubating the patients’ cells with increasing concentrations of rDer p 18 and rDer p 2 (Fig 7C). In three of the four tested patients, rDer p 18 induced a dose-dependent up-regulation of CD203c expression at a concentration of 0.1 μg/ml. Der p 2 induced an up-regulation of CD203c at concentrations between 0.01 and 0.1 μg/ml (Fig 7C).

## Discussion

Our study provides new information regarding the chitinase-like HDM allergen Der p 18. Data regarding the IgE recognition frequency of Der p 18 were highly controversial ranging between 38–63% of HDM-allergic patients [15,17]. In order to re-investigate the IgE recognition frequency in HDM-allergic patients we purified recombinant Der p 18 as folded protein. We assume that the protein is correctly folded because the experimental results from the CD spectrum are in agreement with secondary structure predictions and we obtained similar CD spectra with different rDer p 18 protein preparations. Furthermore we found that rDer p 18 inhibited IgE binding to nDer p 18 completely and showed weak chitin-binding properties indicating that it is biologically active.

The assessment of the IgE binding frequency was performed with a representative number of sera obtained from clinically well characterized HDM-allergic patients who had shown respiratory symptoms attributable to HDM exposure by controlled *in vivo* provocation in the Vienna challenge chamber. Moreover, clinical documentation of HDM-related skin symptoms was available through skin prick testing and recording of symptoms of atopic dermatitis attributable to HDM in the tested patients. Our results show that Der p 18 is recognized by 10% of HDM-allergic patients which is a considerably lower IgE recognition frequency than the one reported earlier but reflects the sensitization rate in a Middle European HDM-allergic population. Similar frequencies of sensitization were also found in larger populations of HDM-allergic patients (n >600 sera from England and France) (data not shown). We have found that certain HDM allergens (e.g., Der p 11) which mainly or exclusively occur in mite bodies but not in feces are more often recognized by HDM-allergic patients with atopic dermatitis. In fact, the IgE recognition rates of Der p 18 were comparable in HDM-allergic patients with only respiratory symptoms and in patients with respiratory symptoms and atopic dermatitis.

It has been reported that Der p 18 is a chitinase-like allergen [17]. Chitin (C_8_H_13_O_5_N)_n_, is a long chain polymeric polysaccharide comprised of N-acetyl-β-D-glucosamine (GlcNAc) residues. It is present in fungal cell walls, the shells and radulae of mollusks and the exoskeleton of crustacean and insects [38]. Chitinases cleave this polymeric structure into simple sugars and can be found in species of all kingdoms (i.e. bacteria, fungi, plants, animals including humans) where they are involved in nutrient digestion, resistance against fungal pathogens, cuticle turnover and immunity [38,39]. Chitinases were also found in humans and there are thought to be involved in certain inflammatory and allergic diseases. Elevated levels of human chitinases (e.g., AMCase) and chitinase-like proteins (e.g., YKL-40) were found in sera and lung tissues of patients suffering from asthma [40,41,42] and it is tempting to speculate that human chitinase-like proteins as well as chitinase-like allergens may have chemotactic activity and perhaps recruit eosinophils and T cells to sites of inflammation [43].

In fact, several chitinase-like allergens have been described, in particular in the context of the latex-fruit syndrome [44,45]. They represent plant defense proteins and belong to the glycoside hydrolase (GH) 19 family, which is characterized by an N-terminal hevein-like domain that shows IgE cross-reactivity with the major latex allergen hevein [45]. Der p 18 belongs to the glycoside hydrolase 18 family which is characterized by an (α/β)_8_-TIM barrel fold structure in the catalytic region [46] and often contains a chitin-binding domain in the C-terminal region [47]. We have developed a structural model of Der p 18 which suggests that the allergen indeed consists of a putative chitinase core domain which however lacks the glutamic acid residue required for chitinase activity and a putative C-terminal chitin-binding domain which are connected by a flexible linker. Interestingly, there seems to be no IgE cross-reactivity between Der p 18 and another chitinase-like HDM allergen, Der p 15 which has a similar architecture and also binds chitin but has low sequence homology to Der p 18 (27%), because IgE reactivity data obtained in our population do not provide evidence for co-sensitization to Der p 18 and Der p 15. Both allergens bound weakly to chitin, which could only be detected with the purified, recombinant allergens but not with the natural allergens in a HDM extract in which natural chitin-binding proteins maybe already bound to chitin. We found that high titre polyclonal anti-Der p 18 antibodies only weakly reacted with Der f 18 in a *D*. *farinae* extract but inhibition experiments indicated some cross-reactivity between Der p 18 and Der f 18.

So far the allergenic activity of Der p 18 has not been studied. We compared rDer p 18 with rDer p 2 for their potential to induce basophil activation in allergic patients and found that rDer p 18 induces basophil activation in an IgE-dependent manner, albeit to a lower extent than Der p 2. This finding together with the fact that we identified a HDM-allergic patient with respiratory and skin symptoms to HDM who was only sensitized to Der p 18 but not to other known HDM allergens, indicates that Der p 18 despite being a minor allergen in terms of IgE recognition frequency has allergenic activity and therefore should be included in diagnostic test panels for HDM allergy.

## Supporting Information

S1 Fig Alignment of the Der p 18 (GenBank accession number: AAY84563.1) amino acid sequence with that of Der p 15 (AAY84564.2). Marked in orange are the chitinase core domain including the conserved cysteines of the catalytic region (C1-C4) and the putative catalytic domains (CD1, CD2: the asterisk indicates the position of the glutamic acid that determines the presence of enzymatic activity). The putative chitin-binding domain (CBD) is highlighted in blue and the region rich in serine, threonine and proline in yellow. Amino acids identical to those of Der p 18 are indicated by dots; dashes represent gaps.
(TIF)

S1 Table Characterization of HDM-allergic patients and HDM allergen-specific IgE levels as determined by ISAC. (TIF)
