# Supplementary figures and images for: Molecular, Structural and Immunological Characterization of Der p 18, a Chitinase-Like House Dust Mite Allergen
Source: PLoS One. 2016 Aug 22;11(8):e0160641. doi: 10.1371/journal.pone.0160641 (PMC4993390; doi:10.1371/journal.pone.0160641)

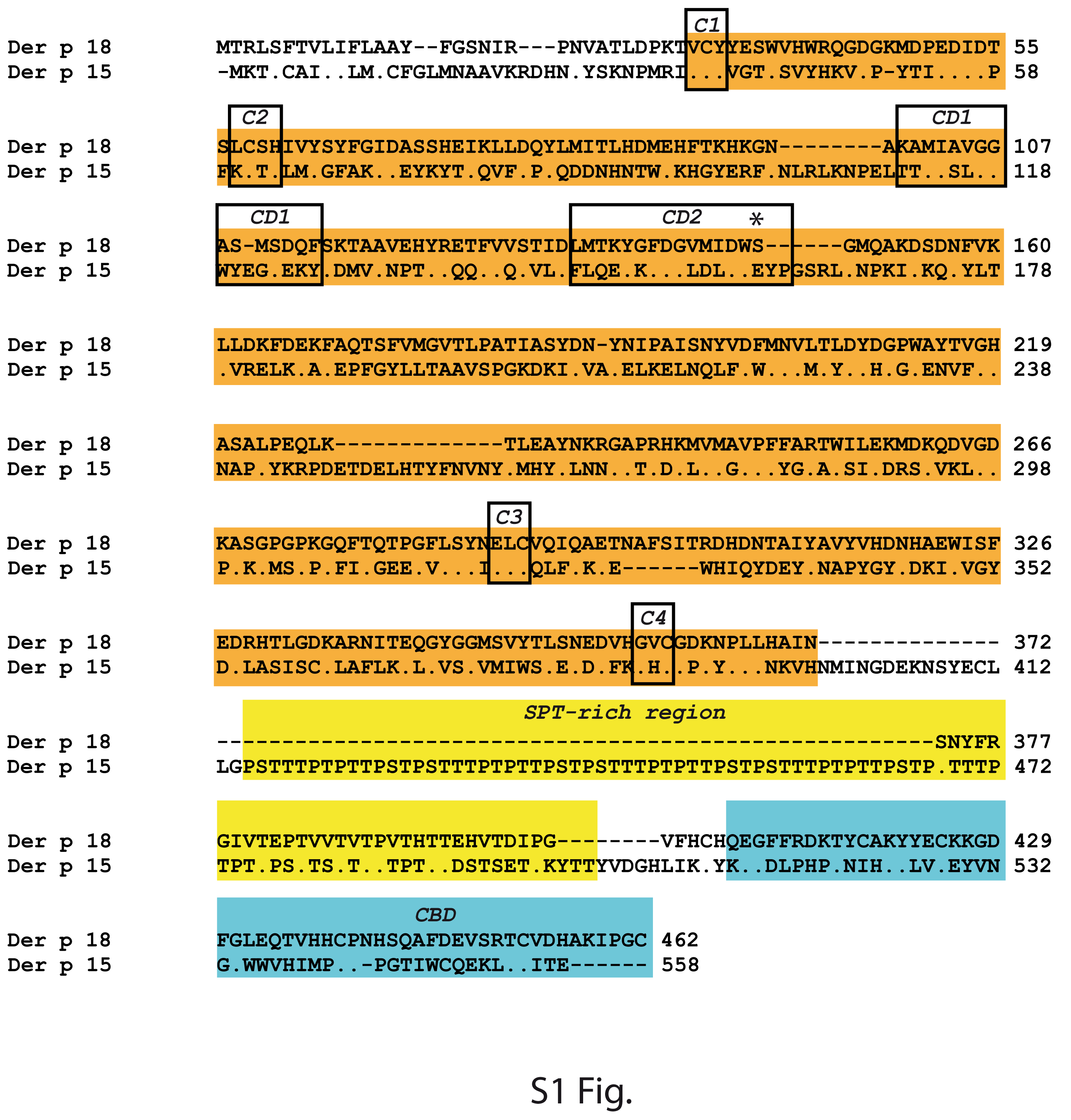

Supplement: S1 Fig — Marked in orange are the chitinase core domain including the conserved cysteines of the catalytic region (C1-C4) and the putative catalytic domains (CD1, CD2: the asterisk indicates the position of the glutamic acid that determines the presence of enzymatic activity). The putative chitin-binding domain (CBD) is highlighted in blue and the region rich in serine, threonine and proline in yellow. Amino acids identical to those of Der p 18 are indicated by dots; dashes represent gaps. (TIF) [file pone.0160641.s001.tif]

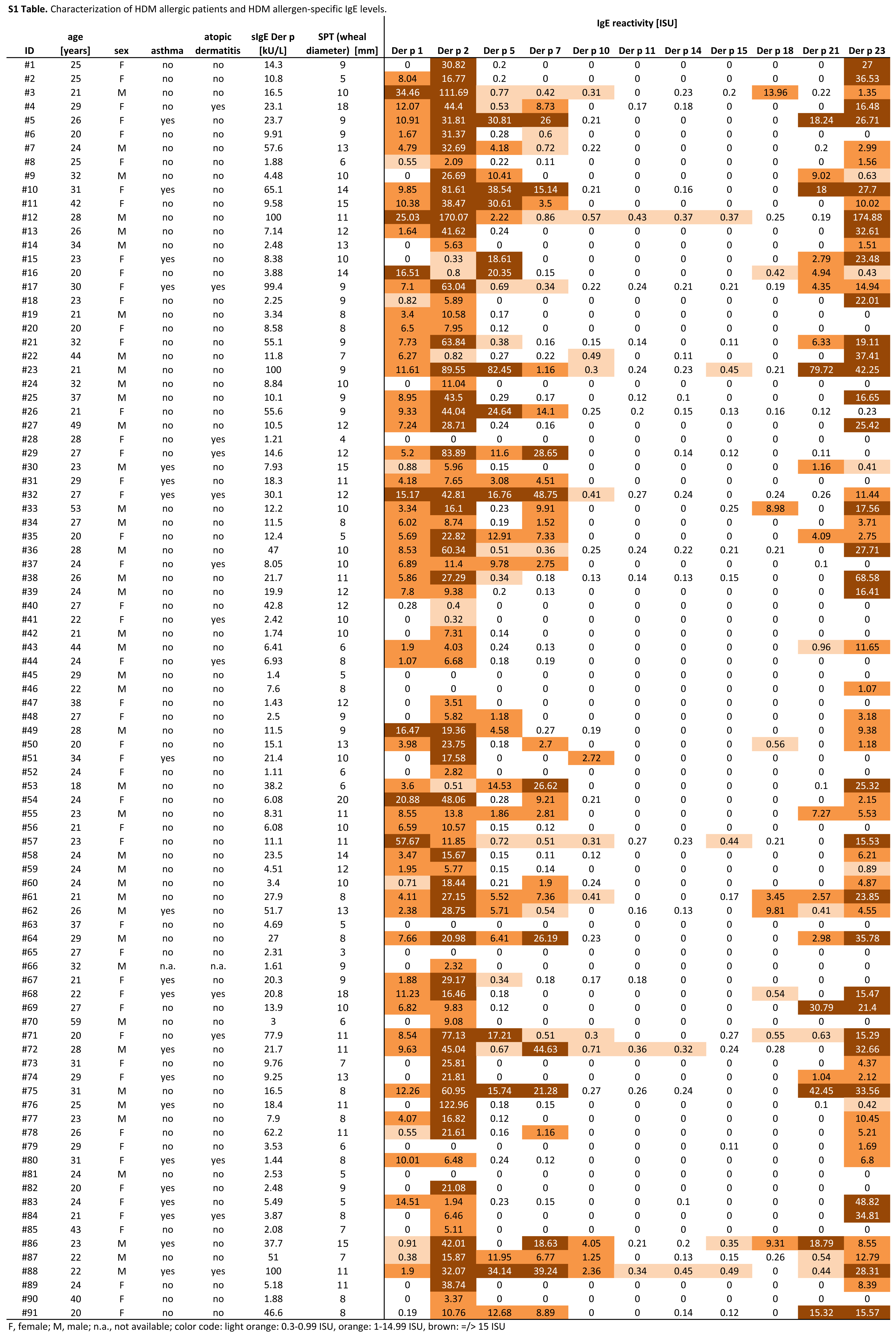

Supplement: S1 Table — (TIF) [file pone.0160641.s002.tif]
